#  Outbreak of Mucormycosis in Coronavirus Disease Patients, Pune, India

**DOI:** 10.3201/eid2801.211636

**Published:** 2022-01

**Authors:** Samir Joshi, Rahul Telang, Muralidhar Tambe, Rajesh Havaldar, Manasi Sane, Afshan Shaikh, Cherry Roy, Kireet Yathati, Sanjaykumar Sonawale, Rupalee Borkar, Rahul Magar, Harshal Bhitkar, Satish Shitole, Leena Nakate, Jyoti Kudrimoti, Vidya Mave

**Affiliations:** Byramjee-Jeejeebhoy Government Medical College and Sassoon General Hospitals, Pune, India (S. Joshi, R. Telang, M. Tambe, R. Havaldar, A. Shaikh, C. Roy, K. Yathati, S. Sonawale, R. Borkar, R. Magar, H. Bhitkar, S. Shitole, L. Nakate, J. Kudrimoti);; Byramjee-Jeejeebhoy Government Medical College—Johns Hopkins University Clinical Research Site, Pune (S. Joshi, M. Sane, V. Mave)

**Keywords:** COVID-19, SARS-CoV-2, coronavirus disease 2019, severe acute respiratory syndrome coronavirus 2, viruses, respiratory infections, zoonoses, invasive mucormycosis, fungal infections, fungi, rhino-sino-orbital mucormycosis, India

## Abstract

We provide an overview of the epidemiology and clinical course of mucormycosis in the coronavirus disease (COVID-19) pandemic era. We conducted a retrospective chart review of 178 patients with clinical or diagnostic, endoscopically or histopathologically confirmed rhino-sino-orbital or cerebral mucormycosis after COVID-19 treatment during the second wave of COVID-19 in Pune, India. Median time to symptom onset from COVID-19 detection was 28 days. Moderate or severe COVID-19 was seen in 73% of patients and diabetes in 74.2%. A total of 52.8% received steroids. Eschar over or inside the nose was seen in 75%, but baseline clinical and laboratory parameters were mostly unremarkable. Bone penetration was present in ≈90% of cases, 30% had soft-tissue swelling of the pterygopalatine fossa and 7% had cavernous sinus thrombosis, and 60% had multifocal mucormycosis. Of the 178 study cases, 151 (85%) underwent surgical debridement. Twenty-six (15%) died, and 16 (62%) of those had multifocal mucormycosis.

As of June 30, 2021, the coronavirus disease (COVID-19) pandemic had affected >182 million persons and resulted in 3.9 million deaths worldwide (*1*). India has reported 30.3 million cases and 398,000 deaths and accounts for the world’s second-largest burden of cases and third-largest burden of deaths (*1*,*2*). Furthermore, more recently, India witnessed the steepest peak of the second wave of COVID-19 of any country that resulted in an unprecedented burden on the health system (*3*). While therapeutics and vaccines were being developed, the national guidelines for COVID-19 management included global standards such as intravenous steroids for moderate and severe COVID-19 and discretionary use of antivirals and antimicrobials by clinicians (*4*). However, during the COVID-19 pandemic, antimicrobial drug use was at an all-time high in India, the consequences of which may include antimicrobial resistance to commonly used drugs, as well as excessive fungal infections (*5*).

Emerging evidence globally suggests varied collateral damage, including post–COVID-19 sequelae such as lung impairment, mental health issues, and thromboembolic events leading to excess illness and death (*6*–*8*). Furthermore, secondary infections with multidrug-resistant organisms and fungi, specifically aspergillus lung infections, have been reported (*9*–*12*). Reports of outbreaks of mucormycosis of the nose and sinuses with subsequent invasion to the orbital and cerebral region among patients successfully treated for COVID-19 have been described in news media in India and in a few case reports (*13*–*17*). Before the COVID-19 pandemic, mucormycosis, caused by filamentous fungus of the order Mucorales, was the second most common invasive fungal infection, associated with high illness and death rates among immunocompromised persons after aspergillosis (*18*–*20*). Of note, India accounted for the world’s largest burden of mucormycosis before the COVID-19 pandemic; several reports have described the clinical course (*21*,*22*). However, data to help identify and define clinical characteristics and outcomes of COVID-19–associated invasive mucormycosis are limited, which is specifically critical for India, a country with a high prevalence of diabetes, which is known to elevate mucormycosis risk (*23*,*24*). We describe demographic, clinical, laboratory, image findings, and outcomes of rhino-sino-orbital and cerebral mucormycosis cluster in an urban tertiary care teaching hospital in western India.

The ethics committee of Byramjee-Jeejeebhoy Government Medical College and Sassoon General Hospitals (BJGMC-SGH) approved this study. Our analysis did not involve an interview or questionnaire, and obtaining individual patient consent was not possible due to the retrospective nature of the study.

## Methods

We conducted a retrospective chart review of patients hospitalized for clinical or histopathologically diagnosed rhino-sino-orbital or cerebral mucormycosis after COVID-19 diagnosis during March 1, 2020–June 5, 2021. These patients were primarily treated in the Medicine and Ear, Nose, Throat departments of BJGMC-SGH, a Maharashtra state–run tertiary care teaching hospital that serves the 5 million low-income and low-middle–income residents of the greater Pune region of Maharashtra state in western India. From the hospital registry at the Ear, Nose, Throat Department of BJGMC-SGH, we also collected the total number of case-patients with rhino-sino-orbital and cerebral mucormycosis during 2016–2019 to compare frequencies of mucormycosis cases before and during the COVID-19 pandemic.

We abstracted data from hospital medical records and hospital medical information systems; data were patient demographics; underlying conditions; and clinical course of COVID-19, including therapeutics, oxygen, or ventilator support. We collected clinical characteristics of mucormycosis, laboratory and radiologic findings including histopathologic results, treatment provided, and clinical outcome–active case (discharged or died) through July 15, 2021.

### Clinical Assessments and Management of Mucormycosis

All patients with suspected mucormycosis underwent clinical assessments and diagnostic naso-sinus endoscopy; if an eschar and surrounding inflammation were seen, a clinical diagnosis of mucormycosis was made. After clinical diagnosis, amphotericin B was initiated intravenously for >21 days. Magnetic resonance imaging (MRI) was done to assess the extent of soft tissue and bone involvement. Within the first 72 hours of clinical diagnosis, surgical debridement was performed if the operating room was available, and tissue was sent in 10% neutral buffered formalin for histopathologic evaluations. Samples were processed using a fully automatic tissue processor. If the tissue received was a bony fragment, it was kept for decalcification in 10% nitric acid, and after satisfactory decalcification, routine hematoxylin and eosin staining was done. Senior pathologists reviewed slides, and whenever necessary, used special stains such as periodic acid Schiff and methanamine silver to highlight fungal hyphae. Any pleomorphic aseptate hyphae with wide-angle branching were identified as a Mucorales group of fungi; differentiation from small, septate hyphae with dichotomous branching was done to rule out other filamentous fungi, such as aspergillus. At discharge, all patients received posaconazole oral medication for >3 more weeks to complete a 6-week antifungal treatment course.

### Definitions

We defined rhino-sino-orbital and cerebral mucormycosis as clinically confirmed (probable) or histopathologically confirmed (proven) mucormycosis. We ascertained underlying illnesses from physician documentation in medical records. We used the definitions of mild, moderate, and severe COVID-19 from the Ministry of Health and Family Welfare for this analysis (*25*). Mild COVID-19 was defined as a positive SARS-CoV-2 infection with or without symptoms and resting oxygen saturation (SpO_2_) >94%. Moderate COVID-19 was defined as the presence of breathlessness, respiratory rate (RR) >24 breaths/min, or SpO2 <93% on room air. Severe COVID-19 was defined as the presence of breathlessness, RR >30 breaths/min, or SpO2 <90% on room air.

To define localized and generalized mucormycosis, we used previously published definitions that were based on findings seen during diagnostic endoscopy and radiologic features on an MRI (*19*,*26*). We defined patients with disease restricted to nasal passages, sinuses, or orbits as having localized disease. If sites affected involved the paranasal sinuses and infiltrated the orbit, we defined patients as having sino-orbital infection. We defined involvement of the paranasal sinuses, orbits, and the brain as generalized rhino-sino-orbital cerebral infection, depending on the extent of the spread. We categorized histologically confirmed diagnosis as proven mucormycosis and clinical and diagnostic endoscopically confirmed diagnosis without histopathologic confirmation as probable mucormycosis.

### Statistical Analysis

We used descriptive statistics to summarize the data. We used the medians and interquartile ranges (IQRs) or means and SDs to describe results as appropriate and described categorical variables as counts and percentages. We used Stata version 15.1 software (StataCorp, https://www.stata.com) for the analysis.

## Results

**Figure 1:**
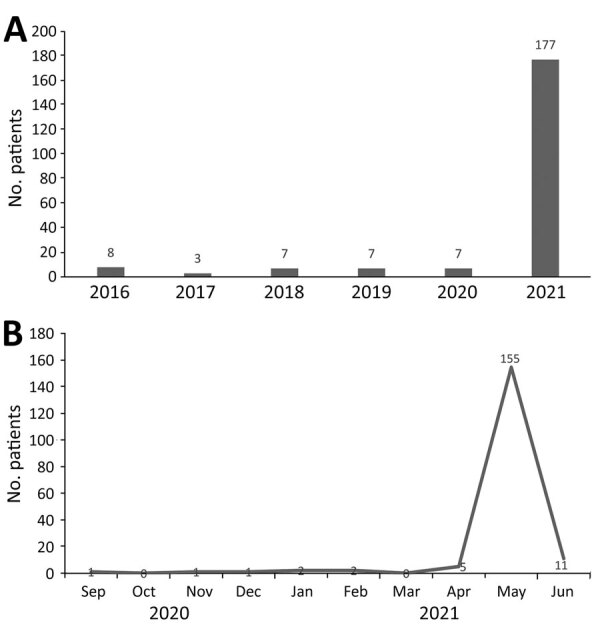
Frequency of rhino-sino-orbital and cerebral mucormycosis cases evaluated and treated at Sassoon General Hospital, Pune, India, before and during coronavirus disease pandemic. A) January 1, 2016–June 14, 2021; B) September 1, 2020–June 14, 2021.

We identified 178 patients who had clinical, endoscopically diagnosed, or histopathologically confirmed localized rhino-, sinus-, orbital-, or rhino-sino-orbital mucormycosis or generalized rhino-sino-orbital-cerebral mucormycosis after completing COVID-19 treatment. During 2016–2020, a range of 3–8 cases of mucormycosis were diagnosed and treated in the Ear, Nose, Throat department of BJGMC-SGH (Figure 1, panel A). In April–May 2021, a total of 160 (90%) cases were diagnosed (Figure 1, panel B).

### Clinical Features

We analyzed the demographic and clinical characteristics of the mucormycosis patients (Table 1). The median age was 51 years (interquartile range [IQR] 42–60 years); of 178 patients, 125 (70%) were men. A total of 132 (74.2%) had previously or newly diagnosed diabetes; 1 patient had HIV infection. We saw moderate or severe COVID-19 in 130 (73%) patients; median length of hospitalization for COVID-19 was 10 days (IQR 8–15 days). Of these, 94 (52.8%) received either oral or intravenous steroids or both; 5 (17%) of mild COVID-19 case-patients received steroids. Ninety-five (98%) of the patients who were treated for COVID-19 received intravenous or oral antimicrobial drugs. Of those who received steroids, 66 (70%) had previously or newly diagnosed diabetes. Of 103 (69%) who were given oxygenation, 5 (4%) needed ventilator support; median duration of oxygenation was 6 days (IQR 3–10 days).

**Table 1:** Clinical characteristics of patients experiencing intensive rhino-sino-orbital and cerebral mucormycosis after undergoing treatment for COVID-19, India

Characteristic	No. case-patients, n = 178
Age	
Age, y, median (range)	51 (42–60)
Age group	
<18	1 (1)
18–45	52 (29)
45–65	103 (58)
>65	22 (12)
Sex	
M	125 (70)
F	53 (30)
Underlying illness	
Diabetes mellitus	
Previously diagnosed	88 (49)
Newly diagnosed	44 (25)
Hypertension	48 (27)
Chronic kidney disease	5 (3)
Cardiomyopathy	4 (2)
Asthma	2 (1)
HIV	1 (1)
COVID-19 characteristics†	
Moderate COVID-19	122 (77)
Mild COVID-19	29 (18)
Severe COVID-19	8 (5)
Hospitalization, d, median (IQR)	10 (8–15)
Required oxygenation	103 (69)
Required ventilator support	5 (4)
Received intravenous steroids	82 (67)
Received oral steroids	12 (17)
Received antimicrobial drugs	95 (98)
Symptoms of mucormycosis	
Facial pain	132 (74)
Headache	96 (54)
Nasal congestion	79 (44)
Nasal discharge	57 (32)
Vision impairment	66 (37)
Time from COVID-19 diagnosis to mucormycosis symptom onset, d (IQR)	28 (15–45)
Vital signs	
Temperature, °F, median (IQR)	98 (98–98.6)
Heart rate >100 beats/min	4 (2)
Heart rate, beats/min, median (IQR)	88 (86–90)
Respiratory rate >20 breaths/min	29 (16)
Respiratory rate, breaths/min, median (IQR)	16 (12–18)
Median BP, systolic, mm Hg	123 (120–128)
Median BP, diastolic, mm Hg	80 (80–86)

*Values are no. (%) patients except as indicated. BP, blood pressure; COVID-19, coronavirus disease; IQR, interquartile range. †These characteristics represent diagnosis and treatment provided for COVID-19 prior to mucormycosis events.

**Figure 2:**
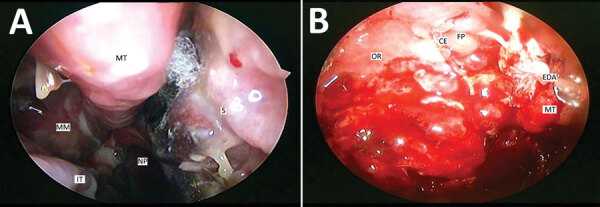
Diagnostic endoscopic examinations of the nasal cavities of 2 patients with mucormycosis after coronavirus disease, Pune, India. A) Right nasal cavity shows crusting in the region of the middle meatus, blackish eschar with fungal elements between the middle turbinate and the nasal septum. Necrosis has begun setting in the part of the nasal septum below the region of the eschar. Mucopurulent discharge is seen trickling from the middle meatus to the nasopharynx because of underlying sinusitis. The inferior turbinate has undergone hypertrophy because of the underlying disease. B) Left nasal cavity shows extradural abscess being drained transnasally. Multiple polyps are noted in the region of the ethmoidal fovea. The crista ethmoidalis, left middle turbinate, and the right orbital roof are destroyed because of the underlying invasion by mucor. CE, crista ethmoidalis; EDA, extradural abscess; FP, ethmoidal fovea; IT, inferior turbinate; MM, middle meatus; MT, middle turbinate; NP, nasopharynx; OR, orbital roof; S, septum.

The median time from COVID-19 detection to mucormycosis symptom onset was 28 days (IQR 15–45 days). The most common symptoms at onset were face pain (74%), headache (54%), and nose pain (48%). Eschar over or in the nose was the symptom that prompted the patient to seek care in 133 (75%) cases; fever was present in 42 (24%) of those patients. In diagnostic endoscopy, 133 (75%) patients had black eschar surrounded by indurated and reddish areas (Figure 2, panel A); 22 (12%) had extradural abscess (Figure 2, panel B). Altered mental status was seen in 17 (10%) patients. Vital signs on admission for mucormycosis were relatively normal (Table 1).

### Laboratory and Radiologic Features

**Figure 3:**
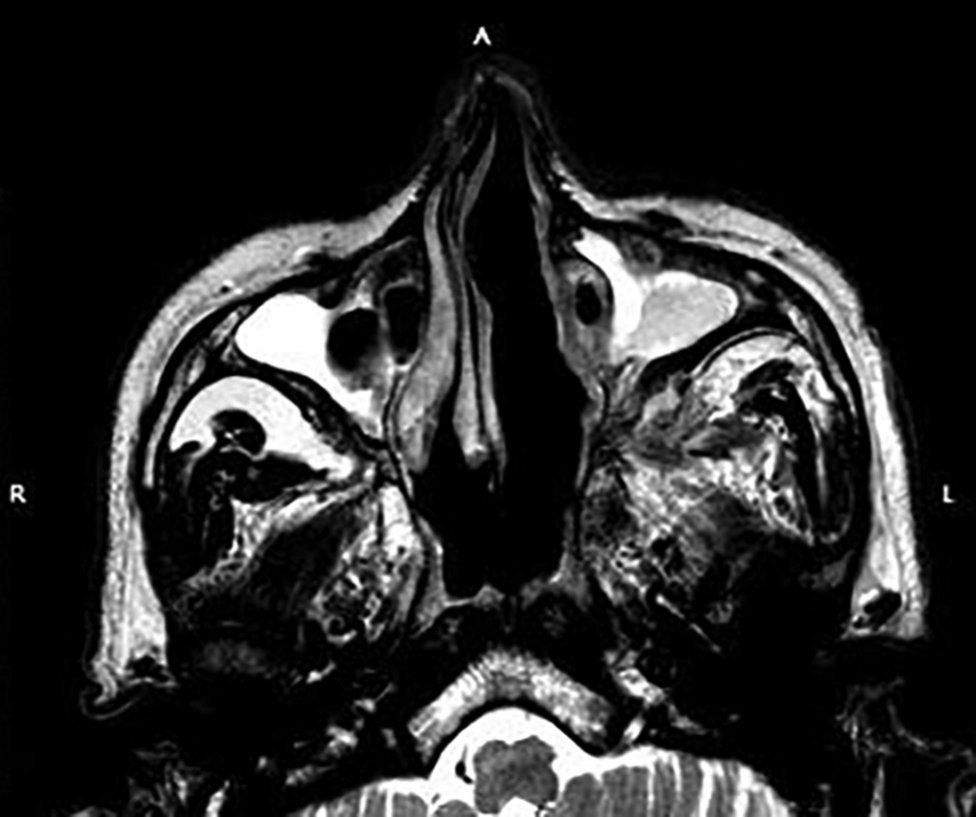
T2-weighted magnetic resonance imaging of the maxillary sinuses of a patient with mucormycosis after coronavirus disease, Pune, India, shows hypointense mucosal thickening bilaterally, more on the left side than the right. Near-complete occlusion of the sinus cavities and obliteration of left osteomeatal unit are seen. There is a mild deviation of the nasal septum with convexity toward the right side. There is mild soft tissue edema with altered signal abnormality involving the left pterygopalatine fossa extending to the left masticator space. L, left; R, right.

At baseline examination, 19 (11%) patients had low hemoglobin at <8dgm/dL, leukocytosis was seen in 50 (28%) patients, and neutropenia (absolute neutrophil count <1,500) in 4 (2%) (Table 2). Swelling of nose and paranasal sinus soft tissue was seen among 172 (97%) patients in MRI (Figure 3). Involvement of the orbital area was seen in 86 (48%) patients and the intracranial area in 22 (12%) cases. Bony penetration was seen in 156 (87.6%) cases (Figure 3) and cavernous sinus thrombosis in 12 (7%) cases.

**Table 2:** Laboratory, histopathologic, and radiologic findings in patients at time of hospital admission for intensive rhino-sino-orbital and cerebral mucormycosis after undergoing treatment for COVID-19, India*

Characteristic	Result
Laboratory findings within 48 h of admission	
Hemoglobin level	10.3 (9.3–11.7)
MCV, fL	82.25 (77.75–86)
MCH, pg	27.6 (26–29)
MCHC, g/dL	32.4 (29.2–33.3)
Lymphocyte counts	
Total cells/mm^3^	8,470 (6,020–12,000)
Lymphocyte, %	18.2 (11.9–24.7)
Distribution	
>10,000 cells/mm^3^	67 (38)
<4,000 cells/mm^3^	15 (8)
Absolute neutrophil count	
<500	0
500–1,000	0
1,000–1,500	4 (2)
1,500–8,000	114 (64)
>8,000	60 (34)
Chemistry parameters	
AST >40 U/L	17 (10)
ALT >40U/L	34 (21)
Platelets, × 10^3^/μL	266.5 (204–336)
Serum creatinine, mg/dL	1 (0.9–1.3)
Potassium, mEq/L	3.54 (2.96–4.05)
Sodium, mEq/L	136 (132–142)
Serum calcium, mg/dL	8.65 (7.95–9.2)
Location(s) involved	
Nose and paranasal sinus	171 (96)
Pterygopalatine fossa	52 (29)
Orbital region	86 (48)
Intracranial soft tissue	22 (12)
Alveolar and palate soft tissue	25 (14)

*Values are no. (%) or median (IQR) except as indicated. ALT, alanine aminotransferase; ANC, absolute neutrophil count; AST, aspartate aminotransferase; COVID-19, coronavirus disease; IQR, interquartile range; MCH, mean corpuscular hemoglobin; MCHC, mean corpuscular hemoglobin concentration; MCV, mean corpuscular volume.

**Figure 4:**
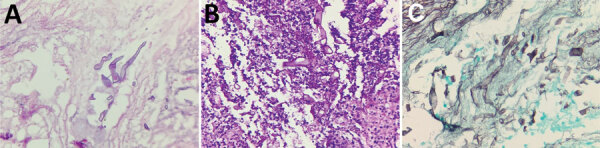
Histopathologic findings for necrotic tissue samples from patients with mucormycosis after coronavirus disease, Pune, India. A) Hematoxylin and eosin stain shows nonpigmented, wide, thin-walled ribbonlike pleomorphic broad aseptate hyphae of mucormycosis (original magnification ×40). B) Periodic acid–Schiff stain shows pleomorphic broad aseptate hyphae of mucormycosis (original magnification ×40). C) Methenamine silver stain shows nonpigmented (hyaline), pauciseptate, ribbonlike hyphae with right-angled branching consistent with Mucorales genera (original magnification ×40).

Fifty-four (30%) case-patients had probable mucormycosis and 124 (70%) had proven mucormycosis; 29 (16%) histopathology results were pending as of the study cutoff date. Histopathologic assessments revealed ribbon-like envelope-shaped aseptate hyphae surrounded by neutrophilic and lymphocytic infiltrations upon staining with hematoxylin and eosin (Figure 4). Pleomorphic broad aseptate hyphae of mucormycosis were seen amidst areas of necrosis.

### Treatment and Outcomes

Seventy-one (40%) patients received a diagnosis of localized mucormycosis disease and 107 (60%) of multifocal mucormycosis (Table 3). Of the 107 with multifocal disease, 47 (44%) had orbital involvement and 22 (12%) had cerebral involvement. Of those with the multifocal affected sites, 76 (83%) had moderate and severe COVID-19 disease, compared with 54 (81%) patients with localized disease (p = 0.83) (Table 3). Of the 178 total mucormycosis patients, 151 (85%) underwent transnasal endoscopic surgical procedure to debride necrotic tissues, 13 (7%) died before surgery, and 13 were scheduled for debridement at the time this report was written. Of all cases, 21 (12%) were treated with intravenous insulin for uncontrolled diabetes or diabetic ketoacidosis during hospitalization. The infusion reactions to amphotericin were not documented. A repeat creatinine was done on day 6 (range day –4 to 6) on 101 (87.8%) of the 115 patients who had initial creatinine test results available. Of those, 14 (13.8%) had elevated creatinine of >25% from the baseline and 6 (5.9%) had >50% of baseline creatinine. As of July 15, 2021, a total of 5 (3%) case-patients were still hospitalized; 147 (82%) had been discharged, and 26 (15%) had died. Of those who died, 16 (62%) had multifocal mucormycosis (Table 3). The median time to death was 15 (IQR 10–27) days. None of the case-patients required extended ventilator support beyond that needed for surgical procedures.

**Table 3:** Sites of mucormycosis in patients with COVID-19 by severity and outcome of illness, Pune, India

Site	COVID-19 severity, no. patients (%)				Outcome, no. patients (%)		
	Mild, n = 29	Moderate, n = 122	Severe, n = 8		Active, n = 5	Discharged, n = 147	Died, n = 26
Unifocal, n = 71							
Localized sinus, n = 71	13 (45)	51 (42)	3 (37)		2 (40)	59 (40)	10 (38)
Multifocal, n = 107							
Generalized, n = 52	5 (18)	37 (30)	3 (37)		3 (60)	38 (26)	11 (42)
Rhino-sinus, n = 6	3 (10)	2 (2)	1 (13)		0	5 (3)	1 (4)
Rhino-orbital, n = 43	6 (21)	30 (25)	1 (13)		0	41 (29)	2 (8)
Rhino-cerebral, n = 2	1 (3)	0	0		0	2 (1)	0
Rhino-orbito-cerebral, n = 4	1 (3)	2 (2)	0		0	2 (1)	2 (8)

## Discussion

We describe illness and death caused by one of the most significant outbreaks of rhino-sino-orbital and cerebral mucormycosis among patients who were undergoing treatment for COVID-19 in a tertiary care teaching hospital in western India. This outbreak coincided with the second wave of the COVID-19 pandemic in India (*1*). In most cases, mucormycosis was diagnosed approximately 1 month after diagnosis of moderate or severe COVID-19 requiring steroids and oxygenation support. Approximately three quarters had either previously or newly diagnosed diabetes, which is known to increase the risk for mucormycosis 2.5-fold (*18*). Furthermore, 12% had diabetic ketoacidosis or uncontrolled diabetes, and >50% of the patients with diabetes received steroids for COVID-19 management. Taken together, the underlying illnesses and medications needed to manage COVID-19 created an ideal setting for the outbreak of mucormycosis (*17*,*23*).

The signs and symptoms of post–COVID-19 mucormycosis in this outbreak were similar to those described previously (*18*,*19*,*26*). The telltale symptoms and signs such as facial pain and headache were more prevalent, and eschar inside the nose was seen in ≈50% of cases. Approximately one tenth of patients had altered mental status. Diagnostic endoscopy and radiological findings showed varied presentations typical for mucormycosis (*18*–*20*). Although the disease is highly invasive in nature, the clinical signs, including vital signs or hematologic or chemistry laboratory parameters, were mostly within reference ranges. Approximately 50% of patients were still receiving treatment as of late July 2021, although only 3% were still hospitalized. The mortality rate may not be notable after discharge from the hospital (*17*); 14.6% of patients died. However, most deaths occurred among those with multifocal disease, underscoring the need for close monitoring for this group (*17*). Drug-related renal impairment known to be associated with amphotericin infusion was lower, similar to prior reports (*27*).

Of note, rhino-sino-orbital or cerebral mucormycosis after COVID-19 treatment had some distinct differences from pre–COVID-19 reports(*28*). In contrast to a previous systematic review in which >75% of the mucormycosis was localized disease, >50% of mucormycosis among case-patients treated for COVID-19 had multiple affected sites (*18*,*19*). Furthermore, bony penetration of the disease among ≈90% of cases, the presence of soft-tissue swelling of the pterygopalatine fossa (located behind the posterior maxillary sinus wall) in ≈30% of cases, and cavernous sinus thrombosis in ≈7% of cases suggest aggressive disease and rapid and extensive local spread of the disease. Among those who completed >21 days of treatment, the proportion who died (14.6%) was lower than the 42% of deaths reported in previous systematic reviews (*18*,*29*). Whether the appropriate surgical interventions and prompt initiation of antifungal treatment optimized the treatment outcomes needs to be investigated and compared with other cohorts.

Potential reasons for the outbreak of mucormycosis during the COVID-19 pandemic need further exploration. India’s 12% diabetes rate among the national population is among the world’s highest, and >14% of its population is prone to develop diabetes from prediabetes (*23*,*24*,*30*). Steroid use for moderate to severe COVID-19 and even for mild COVID-19 may have resulted in uncontrolled diabetes or development of new diabetes, a known risk factor for mucormycosis (*18*). Furthermore, because of a lack of population-level data, the estimated prevalence of mucormycosis in India may be 70-fold higher than global estimates (*21*,*22*,*31*). Thus, the widespread presence of Mucorales fungi in community and hospital settings could become a source of infection in susceptible populations (*32*,*33*). Bhatia (*34*) speculated that oxygenation via facemask or nasal cannula may have inoculated the fungi in the upper respiratory tract when a contaminated water humidifier was used, but further exploration is needed. Moreover, COVID-19–related direct and indirect effects can predispose patients to invasive fungal infection; these effects include virus-induced direct damage to epithelium causing ciliary dysfunction and local immune dysfunction augmenting invasive fungal infection and spread (*35**,**36*). Similarly, virus-related immune suppression can lead to altered immune responses, including decreased T-cell population, which may lead to rapid fungal invasion (*37*).

The first limitation of our study is that, because of its retrospective nature, some data were missing because of a gap in the documentation. Missing data included information on whether the oral steroids were administered in outpatient or inpatient settings for COVID-19, as well as data on status of glycemic control of diabetes. During the outbreak, liposomal amphotericin B was scarce; therefore, most patients received both liposomal and amphotericin B deoxycholate. Documentation of pulmonary involvement among these cases was unavailable. Finally, whether dissemination of mucormycosis took place beyond the rhino-sino-orbital and cerebral regions was not documented. Considering the lower mortality rate, however, dissemination of the disease beyond these regions is unlikely.

By identifying and managing a rhino-sino-orbital and cerebral mucormycosis cluster, we contribute an overview of the epidemiology and clinical course of a devastating fungal infection in the COVID-19 pandemic era. Invasive mucormycosis should be considered among case-patients treated for COVID-19 who experience face pain, nasal blockage, and headache with or without nose eschar in India, particularly among those with diabetes and those who have received steroids. Clinicians should be vigilant about the appropriate use of steroids for COVID-19 management and ensure the use of aseptic precautions during oxygen support to minimize risk for mucormycosis following COVID-19 treatment. Given that India has the world’s second-largest COVID-19 burden (*1*) and the world’s largest estimated Mucorales prevalence (*21*,*22*), the clinical profile and course of the localized, multifocal, or disseminated mucormycosis from other parts of India are critical in helping clinicians detect and manage illness and avert death from invasive mucormycosis.

## References

[1] Dong E, Du H, Gardner L. An interactive web-based dashboard to track COVID-19 in real time. Lancet Infect Dis. 2020;20:533–4. 10.1016/S1473-3099(20)30120-1 32087114 PMC7159018

[2] Ministry of Health and Family Welfare, Government of India. MoHFW dashboard for COVID-19. 2021 [cited 2021 Jun 20]. https://www.mohfw.gov.in

[3] Bhowmick N. How India’s second wave became the worst COVID-19 surge in the world. 2021 [cited 2021 Jun 16]. https://www.nationalgeographic.co.uk/science-and-technology/2021/04/how-indias-second-wave-became-the-worst-covid-19-surge-in-the-world

[4] Ministry of Health and Family Welfare, Government of India; Indian Council of Medical Research COVID-19 National Task Force/Joint Monitoring Group. Clinical guidance for management of adult COVID-19 patients. 2021 [cited 2021 Jun 23]. https://www.icmr.gov.in/pdf/covid/techdoc/COVID_Management_Algorithm_17052021.pdf

[5] Gale J, Shrivastava B. Antibiotics for COVID cases worsen India’s superbug crisis, May 25, 2021 [cited 2021 Jun 23]. https://www.bloomberg.com/news/articles/2021-05-25/antibiotics-for-covid-patients-worsen-india-s-superbug-plight

[6] Nalbandian A, Sehgal K, Gupta A, Madhavan MV, McGroder C, Stevens JS, et al. Post-acute COVID-19 syndrome. Nat Med. 2021;27:601–15. 10.1038/s41591-021-01283-z 33753937 PMC8893149

[7] Pandey P, Agarwal S, Rajkumar. Lung pathology in COVID-19: a systematic review. Int J Appl Basic Med Res. 2020;10:226–33. 10.4103/ijabmr.IJABMR_381_20 33376694 PMC7758785

[8] Silva Junior FJGD, Sales JCES, Monteiro CFS, Costa APC, Campos LRB, Miranda PIG, et al. Impact of COVID-19 pandemic on mental health of young people and adults: a systematic review protocol of observational studies. BMJ Open. 2020;10:e039426. 10.1136/bmjopen-2020-039426 32611746 PMC7358102

[9] Rawson TM, Moore LSP, Zhu N, Ranganathan N, Skolimowska K, Gilchrist M, et al. Bacterial and fungal coinfection in individuals with coronavirus: a rapid review to support COVID-19 antimicrobial prescribing. Clin Infect Dis. 2020;71:2459–68. 10.1093/cid/ciaa530 32358954 PMC7197596

[10] Lansbury L, Lim B, Baskaran V, Lim WS. Co-infections in people with COVID-19: a systematic review and meta-analysis. J Infect. 2020;81:266–75. 10.1016/j.jinf.2020.05.046 32473235 PMC7255350

[11] Lai C-C, Yu W-L. COVID-19 associated with pulmonary aspergillosis: A literature review. J Microbiol Immunol Infect. 2021;54:46–53. 10.1016/j.jmii.2020.09.004 33012653 PMC7513876

[12] Koehler P, Bassetti M, Chakrabarti A, Chen SCA, Colombo AL, Hoenigl M, et al.; European Confederation of Medical Mycology; International Society for Human Animal Mycology; Asia Fungal Working Group; INFOCUS LATAM/ISHAM Working Group; ISHAM Pan Africa Mycology Working Group; European Society for Clinical Microbiology; Infectious Diseases Fungal Infection Study Group; ESCMID Study Group for Infections in Critically Ill Patients; Interregional Association of Clinical Microbiology and Antimicrobial Chemotherapy; Medical Mycology Society of Nigeria; Medical Mycology Society of China Medicine Education Association; Infectious Diseases Working Party of the German Society for Haematology and Medical Oncology; Association of Medical Microbiology; Infectious Disease Canada. Defining and managing COVID-19-associated pulmonary aspergillosis: the 2020 ECMM/ISHAM consensus criteria for research and clinical guidance. Lancet Infect Dis. 2021;21:e149–62. 10.1016/S1473-3099(20)30847-1 33333012 PMC7833078

[13] Mehta S, Pandey A. Rhino-orbital mucormycosis associated with COVID-19. Cureus. 2020;12:e10726. 33145132 10.7759/cureus.10726 PMC7599039

[14] Werthman-Ehrenreich A. Mucormycosis with orbital compartment syndrome in a patient with COVID-19. Am J Emerg Med. 2021;42:264.e5–8. 10.1016/j.ajem.2020.09.032 32972795 PMC7493738

[15] Biswas S. Mucormycosis: the “black fungus” maiming COVID patients in India. 2021 May 9 [cited 2021 Jun 23]. https://www.bbc.com/news/world-asia-india-57027829

[16] Rao S. Bengaluru hospitals see multiple cases of mucor in lungs. 2021 June 24 [cited 2021 Jun 23]. https://timesofindia.indiatimes.com/articleshow/83799233.cms

[17] Rocha ICN, Hasan MM, Goyal S, Patel T, Jain S, Ghosh A, et al. COVID-19 and mucormycosis syndemic: double health threat to a collapsing healthcare system in India. Trop Med Int Health. 2021;26:1016–8. 10.1111/tmi.13641 34117677 PMC8447294

[18] Jeong W, Keighley C, Wolfe R, Lee WL, Slavin MA, Kong DCM, et al. The epidemiology and clinical manifestations of mucormycosis: a systematic review and meta-analysis of case reports. Clin Microbiol Infect. 2019;25:26–34. 10.1016/j.cmi.2018.07.011 30036666

[19] Roden MMZT, Zaoutis TE, Buchanan WL, Knudsen TA, Sarkisova TA, Schaufele RL, et al. Epidemiology and outcome of zygomycosis: a review of 929 reported cases. Clin Infect Dis. 2005;41:634–53. 10.1086/432579 16080086

[20] Sen M, Honavar SG, Bansal R, Sengupta S, Rao R, Kim U, et al.; members of the Collaborative OPAI-IJO Study on Mucormycosis in COVID-19 (COSMIC) Study Group. Epidemiology, clinical profile, management, and outcome of COVID-19-associated rhino-orbital-cerebral mucormycosis in 2826 patients in India - Collaborative OPAI-IJO Study on Mucormycosis in COVID-19 (COSMIC), Report 1. Indian J Ophthalmol. 2021;69:1670–92. 10.4103/ijo.IJO_1565_21 34156034 PMC8374756

[21] Prakash H, Chakrabarti A. Global epidemiology of mucormycosis. J Fungi (Basel). 2019;5:26. 10.3390/jof5010026 30901907 PMC6462913

[22] Chakrabarti A Sr, Singh R. Mucormycosis in India: unique features. Mycoses. 2014;57(Suppl 3):85–90. 10.1111/myc.12243 25187095

[23] Anjana RMDM, Deepa M, Pradeepa R, Mahanta J, Narain K, Das HK, et al.; ICMR–INDIAB Collaborative Study Group. Prevalence of diabetes and prediabetes in 15 states of India: results from the ICMR-INDIAB population-based cross-sectional study. [Erratum in: Lancet Diabetes Endocrinol. 2017;8:e5]. Lancet Diabetes Endocrinol. 2017;5:585–96. 10.1016/S2213-8587(17)30174-2 28601585

[24] Cho NHSJ, Shaw JE, Karuranga S, Huang Y, da Rocha Fernandes JD, Ohlrogge AW, et al. IDF Diabetes Atlas: Global estimates of diabetes prevalence for 2017 and projections for 2045. Diabetes Res Clin Pract. 2018;138:271–81. 10.1016/j.diabres.2018.02.023 29496507

[25] Ministry of Health and Family Welfare, Government of India. Clinical management protocol for COVID-19. 2021 [cited 2021 Sep 16]. https://www.mohfw.gov.in/pdf/UpdatedDetailedClinicalManagementProtocolforCOVID19adultsdated24052021.pdf

[26] Kennedy KJ, Daveson K, Slavin MA, van Hal SJ, Sorrell TC, Lee A, et al.; Australia and New Zealand Mycoses Interest Group of the Australasian Society for Infectious Diseases. Mucormycosis in Australia: contemporary epidemiology and outcomes. Clin Microbiol Infect. 2016;22:775–81. 10.1016/j.cmi.2016.01.005 26806139

[27] Saliba F, Dupont B. Renal impairment and amphotericin B formulations in patients with invasive fungal infections. Med Mycol. 2008;46:97–112. 10.1080/13693780701730469 18324488

[28] Ruan Q, Yang K, Wang W, Jiang L, Song J. Clinical predictors of mortality due to COVID-19 based on an analysis of data of 150 patients from Wuhan, China. [Erratum in: ]. Intensive Care Med. 2020;46:846–8. 10.1007/s00134-020-05991-x 32125452 PMC7080116

[29] Prakash H, Chakrabarti A. Epidemiology of Mucormycosis in India. Microorganisms. 2021;9:523. 10.3390/microorganisms9030523 33806386 PMC8000977

[30] International Diabetes Federation. Diabetes atlas. 9th ed. 2019 [cited 2021 Jun 7]. https://www.diabetesatlas.org/data/en/country/93/in.html

[31] Chakrabarti A, Sood P, Denning D. Estimating fungal infection burden in India: mucormycosis burden as a case study [cited 2021 Sep 16]. https://www.gaffi.org/wp-content/uploads/P1044.pdf

[32] Prakash H, Ghosh AK, Rudramurthy SM, Paul RA, Gupta S, Negi V, et al. The environmental source of emerging *Apophysomyces variabilis* infection in India. Med Mycol. 2016;54:567–75. 10.1093/mmy/myw014 27118802

[33] Prakash H, Singh S, Rudramurthy SM, Singh P, Mehta N, Shaw D, et al. An aero mycological analysis of *Mucormycetes* in indoor and outdoor environments of northern India. Med Mycol. 2020;58:118–23. 10.1093/mmy/myz031 30980083

[34] Bhatia M. The rise of mucormycosis in Covid-19 patients in India. Expert Rev Anti Infect Ther. 2021;1–2. 10.1080/14787210.2021.1960822 34304680

[35] Short KRKJ, Kasper J, van der Aa S, Andeweg AC, Zaaraoui-Boutahar F, Goeijenbier M, et al. Influenza virus damages the alveolar barrier by disrupting epithelial cell tight junctions. Eur Respir J. 2016;47:954–66. 10.1183/13993003.01282-2015 26743480

[36] Herold S, Becker C, Ridge KM, Budinger GR. Influenza virus-induced lung injury: pathogenesis and implications for treatment. Eur Respir J. 2015;45:1463–78. 10.1183/09031936.00186214 25792631

[37] Qin C, Zhou L, Hu Z, Zhang S, Yang S, Tao Y, et al. Dysregulation of immune response in patients with coronavirus 2019 (COVID-19) in Wuhan, China. Clin Infect Dis. 2020;71:762–8. 10.1093/cid/ciaa248 32161940 PMC7108125

